# Discrimination of different varieties of rice in Wuchang area based on *E*-nose and HS-SPME-GC-O-MS

**DOI:** 10.1016/j.fochx.2025.102779

**Published:** 2025-07-10

**Authors:** Lili Qian, Mingming Chen, Yan Song, Tao Zhang, Xingquan Liu, Guoxin Zhou, Hongyan Liu, Feng Zuo

**Affiliations:** a College of Food Science, Heilongjiang Bayi Agricultural University, Daqing 163319, China; b Key Laboratory of Agro-Products Processing and Quality Safety of Heilongjiang province, Daqing 163319, China; c National Coarse Cereals Engineering Research Center, Daqing 163319, China; d National Food and Strategic Reserves Administration, Beijing 100834, China; e College of Food and Health, Zhejiang A & F University, Hangzhou 311300, China; f Institute of Urban Agriculture, Chinese Academy of Agricultural Sciences, National Agricultural Science & Technology Center, Chengdu 610213, China

**Keywords:** Rice, *E*-nose, Headspace solid-phase microextraction-gas chromatography-olfactometry-mass spectrometry, Volatile compounds

## Abstract

This study aimed to discriminate between different varieties of rice from the Wuchang region using a combination of electronic nose (*E*-nose), headspace solid-phase microextraction-gas chromatography-olfactometry-mass spectrometry (HS-SPME-GC-O-MS), and chemometric analysis. Six rice varieties (Wuyoudao No. 4 (WC54), Longyang No. 11 (LY11), ZhongKefa No. 5 (ZKF5), Dongnong No. 425 (DN425), Longyang No. 16 (LY16) and Suijing No. 18 (SJ18)) were analyzed to identify key volatile compounds and aroma-active substances. The electronic nose provided initial differentiation, particularly by the 5 th, 14 th, and 11 th sensors), which showed significant differences in their response strength between ZKF5 and LY16. Furthermore, 148 differential volatile compounds (VIP > 1) were identified by HS-SPME-GC–MS, and aldehydes and alkanes were the most significant contributors to the aroma profiles. Meanwhile, orthogonal partial least squares-discriminant analysis (OPLS-DA) further confirmed the distinctiveness of each rice variety based on their volatile compounds (R^2^Y = 0.991, Q^2^ = 0.900). Additionally, GC-O analysis revealed 36 aroma-active compounds, with WC54 exhibiting the highest aroma intensity, characterized by fruity and creamy notes. The study successfully differentiated WC54 from other varieties, providing a robust method for protecting geographical indication products and ensuring consumer rights.

## Highlights

• *E*-nose technology successfully distinguishes six rice varieties by analyzing aroma profiles.
• HS-SPME-GC–MS identifies 148 key volatile compounds contributing to rice aroma.
• OPLS-DA model validates key volatile markers, enabling precise differentiation of rice varieties.

## Graphical abstract

**Figure:**
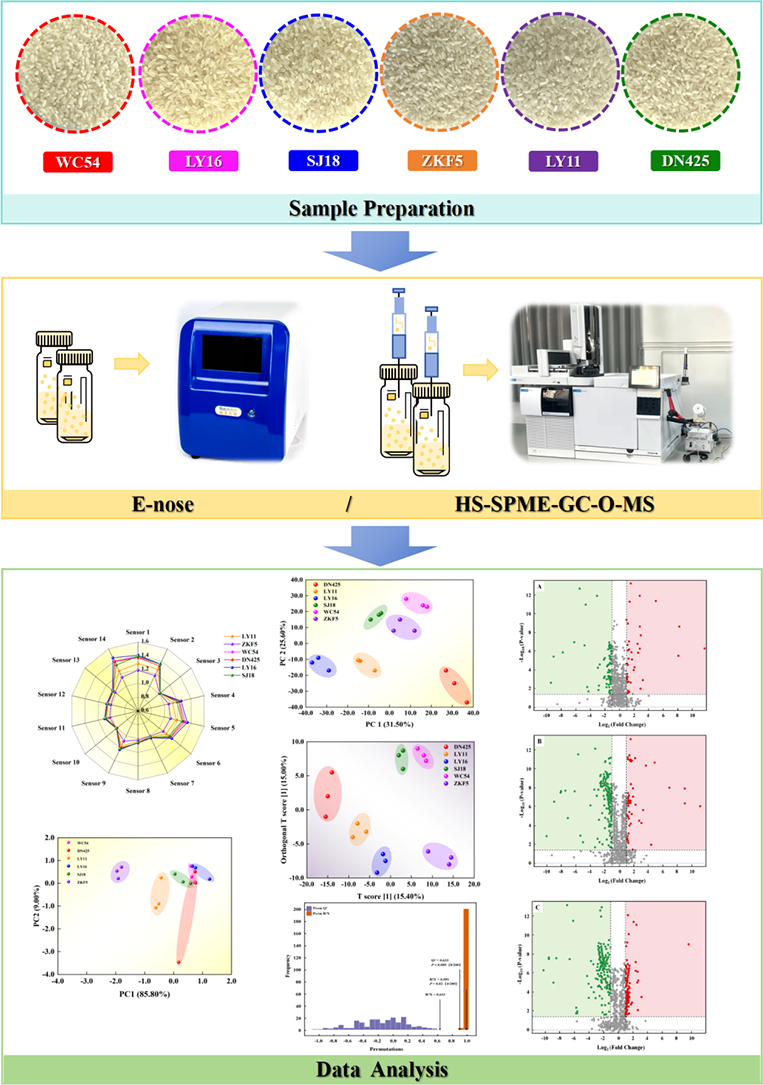

## 1 Introduction

China is the country with the highest rice output and consumption in the world (Amy, 2011). Heilongjiang Province serves as the primary production region for high-quality rice in China, featuring renowned rice brands such as Wuchang rice, Xiangshui rice, and Chahayang rice. Among these, the rice cultivated within the protected area of the Wuchang Geographical Indication stands out as a representative commodity of China's high-value-added agricultural products, possessing significant economic value (Zhang, 2023) However, based on previous field investigation and research, it is found that the prices of different varieties of rice in the Wuchang rice market are different. Based on previous field investigation and research, it is found that the prices of different varieties of rice in the Wuchang rice market are different. In particular, Wuyoudao 4 rice has higher prices than other varieties of rice. As a representative of high-quatily rice in China, Wuyoudao 4 rice is well-known for its moderate grain shape, bright color, rich rice aroma and rich nutrition, and is deeply loved by consumers. It is comparable to internationally renowned rice varieties such as Thai hom rice or Vietnamese moli rice, which are also highly valued for their unique aroma and quality. In addition, Wuyoudao 4 rice has a market situation of oversupply due to its unique growing environment, small planting area, long growing period and relatively limited yield. In recent years, as consumer demand for Wuyoudao 4 rice continues to increase, other rice varieties have appeared on the market and sold at the price and brand of Wuyoudao 4, making it difficult for consumers to distinguish between Wuchang rice varieties (Chen et al., 2023). This not only seriously damages the brand value of Wuyoudao 4 rice, but also infringes on the rights and interests of consumers. Therefore, it is necessary to immediately explore the differences between rice varieties to provide a scientific basis for market supervision and brand protection (Liu et al., 2019).

Electronic nose (*E*-nose) is a kind of odor scanner, its principle is to use a metal oxide and biofilm, according to the small changes in the membrane level caused by the contact of odor molecules to determine whether the smell is strong or weak. It uses specific sensors and pattern recognition systems to quickly provide overall simulated sense of smell information about the sample, indicating the hidden characteristics of the sample. The gas sensor has the characteristics of high sensitivity, reliability and repeatability. At present, *E*-nose has been reported in the quality detection of wines (López de Lerma et al., 2013), edible oil (Xu et al., 2016), meat (Wojnowski et al., 2017), red onion (Russo et al., 2013), apple (Ezhilan et al., 2018) and grains (Olsson et al., 2002). Among them, Xu et al. (2021) used *E*-nose technology to analyze volatile organic compounds (VOCs) during rice aging. The results showed that the E-nose can effectively distinguish rice with different storage times, providing an effective detection method for monitoring the rice aging process. Rahimzadeh et al. (2018) successfully used *E*-nose technology to monitor the changes in volatile organic compounds during rice storage. They can accurately distinguish rice at different storage periods, and are particularly sensitive to the early changes in aroma components of aromatic rice. The classification accuracy rate reaches 100 %, providing an effective method for rice quality control. Zheng et al. (2022) used *E*-nose technology combined with gas chromatography–mass spectrometry (GC–MS) to detect and analyze VOCs in cooked rice. The results showed that the E-nose can quickly and accurately identify the dynamic changes of characteristic flavor substances such as 2-acetyl-1-pyrroline (2-AP) and aldehydes, and effectively distinguish rice samples with different storage conditions and processing techniques. Although research has made progress in rice quality testing, the ability to distinguish similar rice varieties is still insufficient, especially for high-quality rice such as Wuchang Rice. The market is plagued by serious counterfeiting, and existing testing technologies struggle to effectively distinguish between authentic and fake products. However, our research samples exhibit unique quality characteristics—such as moderate grain shape, bright color, and a rich rice aroma—that set them apart from previously studied samples. Therefore, combining electronic nose (*E*-nose) technology with headspace solid-phase microextraction-gas chromatography-olfactometry-mass spectrometry (HS-SPME-GC-O-MS) allows for a more comprehensive analysis of the volatile compounds in rice. This integrated approach offers a more accurate method for identifying the authenticity of Wuchang daohuaxiang rice and provides a scientific basis for addressing the issue of market counterfeiting.

The head space solid phase micro-extraction (HS-SPME) method involves the placement of an SPME fiber in the upper space of the sample and does not require an organic solvent, and set sampling, extraction, and concentration in one step (Delinska et al., 2021; Śliwińska-Bartel et al., 2021). The method used for analyzing high volatile compounds has the advantages of simplicity, convenience, and sensitivity (Liu et al., 2020). Gas chromatography mass spectrometry (GC–MS) is the most important method to identify volatile flavor. However, GC–MS could not determine the contribution of various volatile compounds to overall flavor (Sánchez-Beltrán et al., 2017; Dias et al., 2021a). The gas chromatography olfactory (GC-O) method, in combination with the separation ability of gas chromatography and human olfaction, could evaluate the aromatic active compounds in the composite matrix effectively. Gas chromatography olfactory mass spectrometry (GC-O-MS) can be used to identify volatile flavor compounds and to analyze the contribution of volatile flavor (Ma et al., 2020). Recently, several researchers recognized the geographic origins of rice samples with the utilization of the head space solid phase micro-extraction coupled to gas chromatography mass spectrometry (HS-SPME/GC–MS) strategy simultaneously (Hu et al., 2018; Zhao et al., 2018). Lim et al., (2018a) developed the HS-SPME/GC–MS method to discriminate rice samples from Korea and China with a partial least squares discriminant analysis (PLS-DA) model, and they identified hexanal, 1-hexanol and ten hydrocarbons as discriminatory biomarkers. Nowadays, metabolomics has been successfully applied to investigate metabolites variations in rice among different cultivars, growing regions, and agronomic and storage conditions. The widely accepted and characteristic volatile flavor of seven typical spiced beef was investigated using HS-SPME-GC–MS. A total of 67 volatile flavor compounds were identified in the spiced beef, with 23 compounds in common and 30 aroma-active compounds (Zang et al., 2020). In order to improve the extraction effect of flavor substances and identify the aroma- active substances in fragrant sunflower seed oil, Yin et al. (2023) optimized the extraction conditions of HS-SPME, then the aroma- active substances of fragrant sunflower seed oil were separated and identified by GC-O-MS. A total of 63 volatile substances were identifiedtotally. The results indicated that HS-SPME combined with GC-O-MS could be used to identify the active components of fragrant sunflower seed oil.

In this study, *E*-Nose combined with HS-SPME-GC-O-MS was used to identify volatile flavor substances and key aromatic active substances in six varieties of rice samples in Heilongjiang province. This study aims to establish a discriminant model for the differences between rice varieties by analyzing the key volatile components of different rice varieties in the same region and combining multivariate statistical analysis methods, so as to provide a scientific basis for the identification and protection of rice varieties.

## 2 Materials and methods

### 2.1 Sample collection

Totally six varieties of rice, including Wuyoudao 4 (WC54), Longyang 11 (LY11), ZhongKefa 5 (ZKF5), Dongnong 425 (DN425), Longyang 16 (LY16) and Suijing 18 (SJ18), were collected from Wuchang City, Heilongjiang Province in the year 2022. Five-point field sampling method was used to collect samples, and the samples were then uniformly dried in the laboratory (Chen et al., 2023).

### 2.2 Sample preparation

The rice samples were removed from impurities, selected, hulled by a rice hulling machine to obtain brown rice, and prepared into first-class rice according to GB 1354–2018 “Rice” by a rice hulling machine (VP-32, Yamamoto Corporation, Japan.). The samples were then crushed using a cyclone mill (CT193CyclotecTM, Guangzhou Easy Measurement Instrument Co., Ltd., Guangzhou, China) and sieved through a 60-mesh filter to guarantee homogeneity. The moisture content of the samples was controlled below 14 %, sealed by a self-sealing bag, and stored at 4 °C.

### 2.3 *E*-nose

The rice samples were detected by an electronic nose instrument (cNOSE, Baosheng Technology, Shanghai, China.). The sensor array consists of 14 metal oxide sensors, each with a different sense of a different gas (Table 1). The pretreatment method was according to previous research with some modifications (Qian et al., 2022). Each sample was weighed 10 g and added into the sample bottle, sealed with a headspace method, and sampled three times. The instrument was turned on and preheated for 30 min, and the sensor was selected and gas was washed, the cleaning time was set to 90 s, the sampling time was set to 60 s, and the gas flow rate was set to 1 L/min. The needle is inserted into the sample bottle before sample determination, and the gas is washed between each sample.

**Table 1:** The main response material of *E*-nose sensor.

Sensor	Primary response substance
1	Propane, smoke
2	Alcohol, smoke, isobutane, formaldehyde
3	Ozone
4	Sulfuretted hydrogen
5	Ammonia gas
6	Toluene, acetone, ethanol, hydrogen
7	Methane, natural gas, biogas
8	Liquefied gas
9	Toluene, formaldehyde, benzene, alcohol, acetone
10	Hydrogen
11	Liquefied gas, alkane
12	Liquefied gas, methane
13	Methane
14	Combustible gas, smoke

### 2.4 Hs-SPME

The pretreatment method was according to previous research with some modifications (Zhao et al., 2022). The volatile flavor compounds were extracted using HS-SPME. 3 g of rice powder samples (WC54, LY11, ZKF5, DN425, LY16, SJ18) were accurately weighed, and placed into the headspace bottle (10 mL, Thermo Fisher Shanghai Instrument Co., LTD, Shanghai, China.), screwed the bottle cap with polyethylene diaphragm tightly, and put into the sample tray. The incubation temperature of the sample was 120 °C and the incubation time was 30 min. The volatile components of the sample were extracted in the upper space of the headspace bottle.

### 2.5 GC–MS

The volatile flavor compounds were determined by gas chromatography–mass spectrometry (GC–MS) (Orbitrap Exploris GC Trace 1600, Thermo Fisher Scientific, USA). The method of Dias et al. (2021b)was referred to and appropriately adjusted.

Chromatographic conditions: capillary columns, HP-5MS (30 m × 0.25 mm × 0.25 μm). Column heating program: the oven temperature was programmed maintaining 5 min at 40 °C, then increased to 170 °C at a rate of 10 °C/min maintaining 5 min and then increased at 260 °C at a rate of 10 °C/min maintaining for 2 min. Carrier gas: helium (purity >99.999 %, Chengdu Wangrui Gas Sales Co., Ltd., Chengdu, China) was used as the carrier gas with a flow rate of 1.0 mL/min in a non-split mode.

Mass spectrum conditions: the inlet temperature was 230 °C; the transfer line temperature was set at 260 °C. The ion source was EI, and temperature was 250 °C. The electron energy was 70 eV. The quantity scan range was set to 40–500 *m*/*z* with full scan mode.

Before sample testing, the linear retention index (RI) on HP-5MS was calculated by the retention time of normal alkanes (C6 ∼ C16) and compared with the RI values (Van den Dool & Kratz, 1963) to further ensure the accuracy of each volatile compound.

### 2.6 Identification of volatile compounds

The Mass Spectral Database Network (MSDN) was used for identification of volatile compounds. The volatile compounds were identified by comparing the retention indices (RIs) and searching the mass spectra from the National Institute of Standards and Technology (https://www.nist.gov/). The compounds with a positive and negative matching degree >800 were reported. The RIs of unknown compounds were determined via sample injection with a homologous series of straight-chain alkanes (C_8_ ∼ C_20_). The RI values were reported in the literature, and the data were listed in authentic online databases (http://www.flavornet.org/). The relative contents of the volatile compounds were obtained using the normalization method of peak area.

### 2.7 Identification of aroma profile

An ODP-4 olfactory detector port (ODP-4, GERSTEL, Germany) was used for obtaining the aroma profile. The olfactory detector port was used to acquire aroma characteristics. The interface temperature was set to 200 °C. Wet nitrogen is continuously pumped into the sniffer orifice to prevent the evaluator's nasal cavity from drying out. Before identifying the aroma, the odor was identified using treated samples and standard aroma compounds. The active compounds were recorded as the onset and end time, odor characteristics and intensity of the aroma extracts. The description and identification time of each aromatic active compound was unanimously confirmed by at least two assessors.

### 2.8 Statistical analysis

Analysis of variance with Duncan multiple-range test, principal component analysis (PCA) and orthogonal partial least squares-discriminant analysis (OPLS-DA) were conducted. SPSS (IBM SPSS Statistics 26, IBM, America.) was used for data processing and Origin (Origin 2021b, OriginLab, America.) was used for figure drawing.

## 3 Results and discussion

### 3.1 Difference analysis of aroma components in different varieties of rice based on *E*-nose

**Fig. 1:**
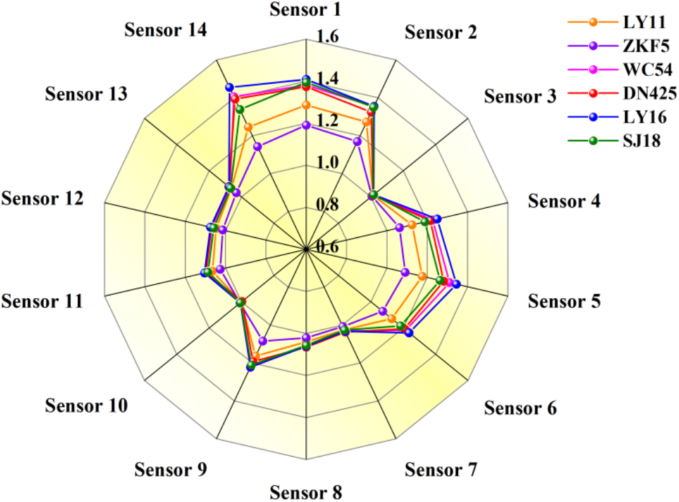
Radar map of different varieties of rice in Wuchang city.

Using the electronic nose data of rice, the radar map is made, which showed that different varieties of rice had different response intensities on 14 sensors (Fig. 1). Specifically, ZKF5 and LY16 had the lowest and highest response intensity on the 5 th sensor (ammonia gas) and 14 th sensor (combustible gas, smoke), respectively. ZKF5 and LY16 had the lowest and highest response intensity on the 11th sensor (alkanes), respectively.

In 7 th sensor (methane, natural gas, biogas), 11 th sensor (liquefied gas, alkane), 12 th sensor (liquefied gas, methane), and 13 th sensor (methane), different varieties of rice (WC54, LY11, ZKF5, DN425, LY16, SJ18) had similar response values overall. In 2 th sensor (alcohol, smoke, isobutane, formaldehyde), 9 th sensor (toluene, formaldehyde, benzene, alcohol, acetone), except for ZKF5, the response values of other varieties of rice were similar. The results showed that the electronic nose data could identify different varieties of rice initially. However, further principal component analysis of the volatile components of different varieties of rice is needed.

### 3.2 Principal component analysis of aroma components in different varieties of rice

E- nose was used to collect volatile information of different varieties of rice, and combined with principal component analysis (PCA), different varieties of rice were identified. The characteristic parameters of the rice sample group were analyzed, and the variance contribution rate (the discriminant factor) and cumulative contribution rate of the sensor variables were obtained. The sum of the two discriminant factors exceeded 94.00 %, indicating that it contained most of the original feature data (Qian et al., 2022).

**Fig. 2:**
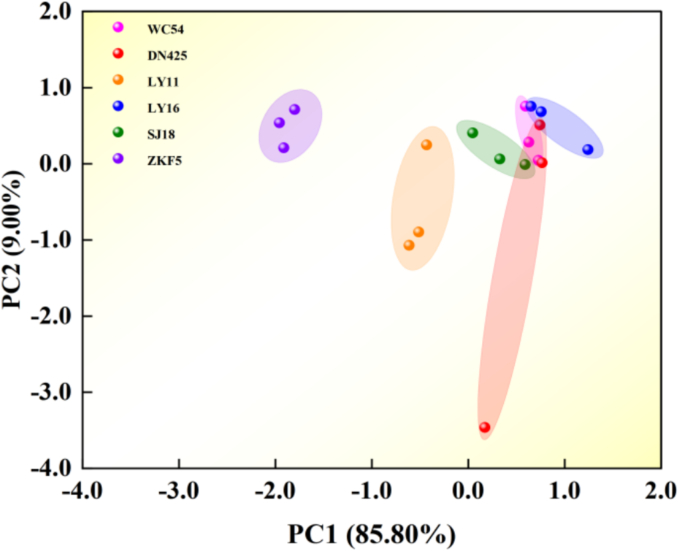
Principal component analysis of different varieties of rice in Wuchang.

As shown in Fig. 2, three rice samples (LY16, SJ18, and ZKF5) were spread across different locations, whereas the other three rice samples (WC54, DN425, and LY16) were overlapped. This suggested that the *E*-nose odor information of different varieties of rice samples was very similarand difficult to be distinguished. Therefore, it is necessary to further detect the specific volatile compounds in different varieties of rice, and analyze the contents and differences of aroma components in them, so as to achieve better classification effect.

### 3.3 Screening of volatile compounds in different varieties of rice based on HS-SPME-GC–MS

#### 3.3.1 Comparative analysis of volatile compounds in different rice varieties

Volatile compounds in different varieties of rice were detected by HS-SPME-GC–MS. Totally 440 compounds were screened, which were divided into alkanes, alcohols, aldehydes, ketones, olefins, acid esters etc. Volatile compounds from different rice varieties were screened based on orthogonal partial least squares discriminant analysis (OPLS-DA) combined with peak area, retention index, variable importance projection (VIP) and *P*-value (Beleggia et al., 2011; Xia & Wishart, 2011). As shown in Table 2, among the 148 differentially expressed volatile compounds, there were 40 alcohols, 30 aldehydes, 26 alcohols, 17 ketones, 8 olefins, 13 acid esters, and 14 other substances. Among them, different varieties of rice had great differences in volatile components. For example, 75 volatile components such as Tridecane, Tetradecane, Pentadecane, Hexanal, Heptanal, Nonanal, Decanal, Ethanol, Ethylene glycol, 3-octen-2-ol, 1-Nonen-4-ol, Cyclobutyl phenyl ketone, Ethanedione, (4-methylphenyl)phenyl-, Sulfurous acid, butyl nonyl ester were detected in 6 varieties of rice at the same time; 13 volatile components such as (*E*)-Oct-3-en-2-one、Phytone、Butyric acid, 2-phenyl-, dec-2-yl ester、4-Methylbenzyl salicylate、Phenol、2-acetyl-1-pyrroline were detected in 5 varieties of rice at the same time; some volatile components are substances unique to certain rice varieties, such as 1-Ethyl-3-vinyl-adamantane, which was only detected in WC54 and DN425. 3-Phenylpropionic acid and 4-cyanophenyl estere were only detected in WC54.

**Table 2:** The volatile compounds with significant differences in different rice samples.

No.	Volatile compounds	CAS	RI	VIP	Relative content (%)					
					WC54	LY11	LY16	DN425	ZKF5	SJ18
**Alkanes (40)**										
1	Spiro[bicyclo[3.1.0]hexane-2,1′-cyclopropane]	N/A	1269	1.50	3.87 ± 0.02^a^	0.31 ± 0.01^b^	0.18 ± 0.01^c^	0.34 ± 0.02^b^	2.13 ± 0.03^a^	0.08 ± 0.21^d^
2	Silane, dimethyl(dimethyl(3,5-difluorophenoxy)silyloxy)pentadecyloxy-	N/A	1350	1.59	2.87 ± 0.12^a^	0.21 ± 0.02^d^	0.20 ± 0.02^d^	0.43 ± 0.03^c^	1.13 ± 0.06^b^	0.05 ± 0.33^e^
3	Silane, dimethyl(dimethyl(2-chlorophenoxy)silyloxy)octadecyloxy-	N/A	1419	1.51	0.67 ± 0.05^a^	0.05 ± 0.01^cd^	0.06 ± 0.02^cd^	0.08 ± 0.01^c^	0.08 ± 0.01^c^	0.24 ± 0.02^b^
4	Hexasiloxane, tetradecamethyl-	107–52-8	1437	1.86	0.44 ± 0.02^a^	–	–	–	–	0.28 ± 0.03^b^
5	Cyclopentane, ethylidene-	2146-37-4	1522	1.52	0.18 ± 0.01^a^	–	–	–	0.02 ± 0.01^b^	–
6	1,1,3,3-Tetramethyl-1,3-di-n-propyldisiloxane	18,001–73-5	1525	1.53	0.30 ± 0.02^a^	–	–	0.11 ± 0.01^b^	–	0.17 ± 0.02^b^
7	Decane, 6-ethyl-2-methyl-	62,108–21-8	1450	1.17	0.49 ± 0.04^c^	0.83 ± 0.03^a^	0.64 ± 0.06^b^	0.06 ± 0.01^d^	0.87 ± 0.05^a^	0.46 ± 0.08^c^
8	1,3,5,7,9,11-Hexaethyl-5-oxy-9-butoxytricyclo[5.5.1.1(3,11)]hexasiloxane	N/A	1585	1.49	1.33 ± 0.23^d^	1.66 ± 0.11^b^	2.60 ± 0.12^a^	1.49 ± 0.06^c^	1.65 ± 0.09^b^	1.26 ± 0.18^de^
9	Tetracyclo[5.2.1.0(2,6)0.0(3,5)]decane, 4,4-dimethyl-	74,646–38-1	1602	1.41	0.09 ± 0.02^b^	–	–	0.13 ± 0.01^a^	0.12 ± 0.01^a^	–
10	Cyclopentane, 1,3-dichloro-, trans-	26,688–50-6	1628	1.94	0.53 ± 0.05^a^	0.06 ± 0.01^c^	0.14 ± 0.03^b^	0.17 ± 0.02^b^	0.19 ± 0.03^b^	0.67 ± 0.08^a^
11	Decane, 2,6,8-trimethyl-	62,108–26-3	1511	1.54	–	–	0.24 ± 0.03^b^	–	0.05 ± 0.01^c^	0.34 ± 0.09^a^
12	1-Bromo-2-(4-hydroxyphenyl)ethane	N/A	1684	1.44	0.55 ± 0.02^a^	0.39 ± 0.03^b^	0.34 ± 0.06^b^	–	–	–
13	Octane, 3-ethyl-2,7-dimethyl-	62,183–55-5	1695	1.45	0.59 ± 0.02^a^	0.17 ± 0.04^c^	0.16 ± 0.02^c^	0.30 ± 0.03^ab^	0.27 ± 0.01^ab^	0.57 ± 0.08^a^
14	Bicyclo[2.2.1]heptane-2-carboxaldehyde, 3-methyl-, (2-endo,3-exo)-	15,780–36-6	1708	1.19	0.44 ± 0.02^a^	0.12 ± 0.05^c^	0.15 ± 0.03^c^	0.05 ± 0.00^d^	0.28 ± 0.02^b^	0.12 ± 0.00^c^
15	Hexane, 3,4-bis(1,1-dimethylethyl)-2,2,5,5-tetramethyl-	62,850–21-9	1368	1.21	0.43 ± 0.02^a^	0.11 ± 0.05^c^	0.14 ± 0.03^c^	0.04 ± 0.00^d^	0.27 ± 0.02^b^	0.11 ± 0.00^c^
16	Bicyclo[3.1.1]heptane, 6,6-dimethyl-2-methylene-, (1*S*)-	18,172–67-3	1744	1.28	1.45 ± 0.16^a^	0.17 ± 0.03^c^	–	–	0.29 ± 0.03^b^	0.18 ± 0.06^c^
17	Heptasiloxane, hexadecamethyl-	541–01–5	1602	1.08	0.06 ± 0.01^b^	–	0.07 ± 0.02^b^	0.17 ± 0.02^a^	0.06 ± 0.01^b^	–
18	5,5-Dimethyl-1-vinylbicyclo[2.1.1]hexane	16,626–39-4	1774	1.67	0.12 ± 0.02^cd^	0.13 ± 0.03^cd^	0.14 ± 0.02^cd^	0.19 ± 0.02^c^	0.28 ± 0.03^b^	0.37 ± 0.09^a^
19	Cyclopentane, 1-methyl-2-methylene-	41,158–41-2	1784	1.37	0.19 ± 0.04^b^	0.13 ± 0.03^c^	0.17 ± 0.03^b^	0.38 ± 0.07^a^	0.10 ± 0.02^d^	0.24 ± 0.06^ab^
20	Cyclopropane, 1,2-dimethyl-3-methylene-, cis-	4866-55-1	1859	1.06	0.09 ± 0.02^b^	–	0.02 ± 0.01^c^	0.14 ± 0.03^a^	–	0.10 ± 0.05^ab^
21	Cyclohexane, 1-methyl-3-propyl-	4291-80-9	1661	1.01	0.09 ± 0.01^b^	–	0.17 ± 0.03^a^	0.09 ± 0.03^b^	0.09 ± 0.02^b^	0.12 ± 0.05^a^
22	Octane, 2,2,6-trimethyl-	62,016–28-8	1735	1.34	0.02 ± 0.01^a^	–	–	0.02 ± 0.01^a^	–	–
23	Difluoromesityl(2,4,6-tri-*tert*-butylphenyl)silane	N/A	2048	1.29	0.04 ± 0.02^bc^	0.08 ± 0.02^b^	0.04 ± 0.01^bc^	0.17 ± 0.06^a^	0.02 ± 0.00^c^	0.03 ± 0.01^c^
24	Silane, diethylisobutoxypropoxy-	N/A	2195	1.50	0.03 ± 0.01^bc^	0.03 ± 0.01^bc^	0.04 ± 0.01^b^	0.10 ± 0.03^d^	0.53 ± 0.01^b^	1.02 ± 0.01^a^
25	1,2,4,5-Tetroxane, 3,3,6,6-tetraphenyl-	16,204–36-7	2205	1.96	0.02 ± 0.00^cd^	0.02 ± 0.04^cd^	0.04 ± 0.04^cd^	0.08 ± 0.02^c^	0.68 ± 0.01^b^	1.12 ± 0.04^a^
26	1-Ethyl-3-vinyl-adamantane	N/A	2112	1.57	0.07 ± 0.01^a^	–	–	0.07 ± 0.01^a^	–	–
27	1,1,1,5,7,7,7-Heptamethyl-3,3-bis(trimethylsiloxy)tetrasiloxane	38,147–00-1	1848	1.39	0.04 ± 0.02^ab^	–	0.03 ± 0.01^ab^	0.13 ± 0.03^a^	–	0.13 ± 0.05^a^
28	Tridecane	629–50-5	2507	1.63	6.92 ± 0.11^a^	0.95 ± 0.08^b^	0.77 ± 0.05^b^	0.47 ± 0.05^c^	0.22 ± 0.08^cd^	0.12 ± 0.03^d^
29	Tetradecane	629–59-4	2538	1.60	1.67 ± 0.04^c^	1.97 ± 0.11^b^	1.45 ± 0.06^cd^	1.69 ± 0.04^c^	2.40 ± 0.23^b^	3.51 ± 0.35^a^
30	Pentadecane	629–62-9	2654	1.17	0.42 ± 0.04^d^	2.37 ± 0.08^ab^	0.45 ± 0.05^d^	1.86 ± 0.05^bc^	3.13 ± 0.09^a^	2.54 ± 0.26^ab^
31	Pristane	1921-70-6	2661	1.00	0.47 ± 0.07^b^	0.29 ± 0.08^bc^	0.25 ± 0.04^bc^	0.68 ± 0.04^a^	0.64 ± 0.00^a^	0.16 ± 0.02^d^
32	4-Methylhexadecane	25,117–26-4	2681	1.56	0.18 ± 0.03^b^	0.20 ± 0.03^ab^	0.25 ± 0.02^a^	0.25 ± 0.02^a^	0.06 ± 0.01^c^	–
33	6-Methyloctadecane	10,544–96-4	2724	1.55	0.03 ± 0.00^c^	0.09 ± 0.04^b^	0.20 ± 0.04^a^	0.10 ± 0.02^b^	0.16 ± 0.01^a^	0.11 ± 0.04^ab^
34	4-Ethyl-tetradecane	55,045–14-2	2735	1.21	0.46 ± 0.05^b^	0.33 ± 0.05^bc^	0.23 ± 0.02^bc^	0.64 ± 0.06^a^	0.35 ± 0.02^bc^	0.54 ± 0.08^ab^
35	Undecane,4-Methyl-	2980-69-0	2230	1.38	–	–	–	–	0.26 ± 0.03^a^	–
36	4-Ethyldecane	1636-44-8	2463	1.07	0.11 ± 0.01^a^	–	–	0.06 ± 0.01^b^	–	–
37	Cyclotetradecane	295–17-0	2489	1.58	–	–	0.27 ± 0.02^bc^	0.46 ± 0.07^ab^	–	0.70 ± 0.20^a^
38	Nonylcyclohexane	2883-02-5	2651	1.05	0.19 ± 0.02^b^	0.34 ± 0.03^a^	0.20 ± 0.02^b^	0.05 ± 0.00^c^	0.29 ± 0.01^a^	0.28 ± 0.04^ab^
39	3-Methylpentadecane	2882–96-4	2342	1.14	0.41 ± 0.04^bc^	0.55 ± 0.08^b^	0.48 ± 0.05^b^	0.86 ± 0.04^a^	0.76 ± 0.02^ab^	1.00 ± 0.09^a^
40	2,6,10-Trimethylpentadecane	3892-00-0	2744	1.23	0.10 ± 0.03^bc^	0.22 ± 0.03^b^	0.14 ± 0.03^bc^	0.48 ± 0.02^a^	0.11 ± 0.01^bc^	0.05 ± 0.00^c^
**Aldehydes (30)**										
1	E-2-Methyl-5-(fur-3-yl)-pent-2-enal	N/A	1250	1.67	0.64 ± 0.12^b^	0.65 ± 0.04^b^	0.55 ± 0.03^bc^	0.58 ± 0.02^bc^	0.98 ± 0.03^a^	0.54 ± 0.02^bc^
2	4-Pentenal, 2-ethyl-	5204-80-8	1501	1.60	1.95 ± 0.21^a^	1.45 ± 0.08^ab^	0.63 ± 0.06^cd^	0.60 ± 0.01^cd^	0.05 ± 0.01^e^	0.78 ± 0.03^c^
3	Bicyclo[2.2.1]heptane-2-carboxaldehyde, *exo*-	3574-55-8	1502	1.77	0.68 ± 0.04^cd^	1.18 ± 0.23^b^	1.01 ± 0.05^bc^	1.05 ± 0.05^bc^	0.95 ± 0.02^c^	1.60 ± 0.05^a^
4	Acetaldehyde butyl isopropyl acetal	N/A	1767	1.78	1.78 ± 0.09^a^	1.64 ± 0.05^a^	0.40 ± 0.02^b^	0.37 ± 0.02^b^	0.26 ± 0.02^bc^	1.58 ± 0.03^a^
5	Hexanal, 5,5-dimethyl-	55,320–58-6	1800	1.46	0.49 ± 0.02^cd^	0.63 ± 0.05^c^	0.81 ± 0.02^b^	0.91 ± 0.01^ab^	1.02 ± 0.04^a^	0.69 ± 0.01^c^
6	Alpha.-Butylcinnamaldehyde	N/A	2392	1.11	1.67 ± 0.08^ab^	1.55 ± 0.04^bc^	1.40 ± 0.03^cd^	1.70 ± 0.54^a^	1.65 ± 0.08^ab^	1.47 ± 0.08^cd^
7	3,5-di-tert-Butyl-4-hydroxybenzaldehyde	1620-98-0	2534	1.25	1.89 ± 0.11^ab^	1.98 ± 0.12^a^	1.20 ± 0.04^c^	0.80 ± 0.02^cd^	1.30 ± 0.04^c^	1.98 ± 0.10^a^
8	Hexanal	66–25-1	1296	1.78	19.11 ± 0.12^a^	19.49 ± 0.77^a^	17.33 ± 0.17^ab^	16.40 ± 0.30^c^	18.34 ± 0.55^ab^	17.18 ± 0.34^ab^
9	Heptanal	111–71-7	1411	1.70	0.78 ± 0.06^cd^	1.28 ± 0.06^b^	1.69 ± 0.08^a^	1.09 ± 0.12^c^	1.01 ± 0.04^c^	0.82 ± 0.10^cd^
10	Nonanal	124–19-6	1568	2.08	17.31 ± 0.08^d^	24.16 ± 0.51^b^	16.84 ± 0.23^d^	36.96 ± 0.27^a^	23.73 ± 0.42^bc^	25.54 ± 0.30^b^
11	Decanal	112–31-2	1693	1.98	2.23 ± 0.06^a^	1.73 ± 0.06^bc^	0.09 ± 0.01^e^	1.49 ± 0.05^cd^	1.87 ± 0.10^ab^	1.24 ± 0.03^bc^
12	Isovaleraldehyde	590–86-3	1575	1.72	0.07 ± 0.02^b^	0.07 ± 0.02^b^	0.09 ± 0.03^b^	0.09 ± 0.03^b^	1.08 ± 0.15^a^	0.07 ± 0.03^b^
13	2-Methylbutanal	96–17-3	1163	1.37	0.13 ± 0.04^b^	0.10 ± 0.01^b^	0.11 ± 0.02^b^	0.07 ± 0.02^c^	0.23 ± 0.03^a^	0.11 ± 0.04^b^
14	Valeraldehyde	110–62-3	1680	1.58	0.31 ± 0.03^b^	0.60 ± 0.31^c^	1.00 ± 0.07^a^	0.45 ± 0.03^b^	–	–
15	2-hexen-1-al	505–57-7	1722	1.66	–	0.08 ± 0.00^ab^	0.05 ± 0.01^b^	–	–	–
16	2-Heptenal	57,266–86-1	1729	1.43	0.43 ± 0.01^b^	0.10 ± 0.09^c^	0.98 ± 0.05^a^	0.49 ± 0.01^b^	0.46 ± 0.01^b^	1.13 ± 0.12^a^
17	Benzaldehyde	100–52-7	1734	1.25	0.63 ± 0.05^c^	0.43 ± 0.08^d^	0.72 ± 0.04^ab^	0.70 ± 0.06^ab^	0.85 ± 0.04^a^	0.71 ± 0.06^ab^
18	Octanal	124–13-0	1778	1.37	1.52 ± 0.03^c^	2.74 ± 0.13^a^	2.66 ± 0.01^a^	1.91 ± 0.05^b^	1.41 ± 0.03^cd^	1.54 ± 0.14^c^
19	(2*E*)-2-Decenal	3913-81-3	1365	1.45	0.27 ± 0.03^a^	0.11 ± 0.00^bc^	0.10 ± 0.02^bc^	0.12 ± 0.02^bc^	0.19 ± 0.00^b^	0.14 ± 0.01^bc^
20	(2E)-2-Octenal	2548-87-0	1859	1.04	0.59 ± 0.05^c^	0.05 ± 0.00^de^	2.64 ± 0.07^a^	0.77 ± 0.07^d^	0.05 ± 0.00^de^	1.58 ± 0.10^b^
21	(E)-Citral	141–27-5	1994	1.43	–	–	–	0.07 ± 0.01^ab^	0.16 ± 0.01^a^	–
22	2-Isopropenyl-5-methylhex-4-enal	75,697–98-2	2175	1.17	0.05 ± 0.00^ab^	0.06 ± 0.01^ab^	–	–	–	0.15 ± 0.02^a^
23	(2E)-2-Nonenal	18,829–56-6	2195	1.61	0.56 ± 0.07^d^	1.37 ± 0.30^bc^	1.56 ± 0.04^b^	1.92 ± 0.02^a^	0.58 ± 0.05^d^	1.03 ± 0.11^c^
24	2,3-expoxycitral	16,996–12-6	2223	1.55	–	–	–	0.42 ± 0.04^a^	0.14 ± 0.01^b^	0.22 ± 0.01^b^
25	Nona-2,4-dien-1-al	6750-03-4	2282	1.85	–	0.23 ± 0.05^b^	0.03 ± 0.02^c^	–	0.12 ± 0.01^ab^	–
26	(Z)-2-decen-1-al	2497-25-8	2303	1.24	0.17 ± 0.04^d^	0.35 ± 0.07^b^	1.33 ± 0.09^a^	0.22 ± 0.01^bc^	0.13 ± 0.01^d^	0.22 ± 0.02^bc^
27	Undecanal	112–44-7	2319	1.05	0.52 ± 0.02^a^	0.31 ± 0.04^bc^	0.54 ± 0.05^a^	0.24 ± 0.02^bc^	0.39 ± 0.03^b^	0.38 ± 0.05^b^
28	Undecenal	2463-77-6	2334	1.27	0.36 ± 0.01^ab^	0.32 ± 0.04^ab^	0.42 ± 0.05^a^	0.25 ± 0.02^bc^	–	–
29	Lauryl aldehyde	112–54-9	2408	1.02	0.13 ± 0.03^d^	0.23 ± 0.03^b^	0.30 ± 0.03^b^	0.14 ± 0.02^d^	0.18 ± 0.03^bc^	0.54 ± 0.06^a^
30	Tetradecanal	124–25-4	2427	1.41	0.08 ± 0.03^a^	–	–	–	–	–
**Alcohols (26)**										
1	p-Menth-1(7)-en-9-ol	29,548–16-1	1227	1.34	0.08 ± 0.01^b^	0.19 ± 0.05^a^	0.06 ± 0.02^b^	–	–	–
2	Cyclohexanemethanol, 4-methylene-	1004-24-6	1359	2.01	–	0.27 ± 0.01^a^	–	0.28 ± 0.03^a^	–	–
3	2-Methyl-1-phenylbut-3-en-1-ol	25,201–44-9	1729	1.74	0.12 ± 0.03^a^	–	–	0.05 ± 0.01^ab^	0.10 ± 0.02^a^	0.06 ± 0.02^ab^
4	2,4,4-Trimethyl-1-pentanol, trifluoroacetate	N/A	1811	1.04	0.40 ± 0.04^b^	0.61 ± 0.05^ab^	0.98 ± 0.07^a^	0.25 ± 0.03^c^	0.56 ± 0.06^ab^	0.43 ± 0.06^b^
5	5,10-Pentadecadiyn-1-ol	64,275–50-9	2023	2.01	0.65 ± 0.06^a^	0.29 ± 0.01^b^	0.33 ± 0.03^b^	0.29 ± 0.01^b^	0.29 ± 0.02^b^	0.19 ± 0.02^c^
6	Dihydro-cis-.alpha.-copaene-8-ol	58,569–27-0	1708	1.08	1.58 ± 0.12^c^	6.64 ± 0.45^a^	4.15 ± 0.12^b^	1.01 ± 0.02^cd^	2.44 ± 0.14^bc^	2.86 ± 0.14^bc^
7	3,6-Nonadien-1-ol, (E,Z)-	56,805–23-3	2341	1.53	–	0.06 ± 0.00^ab^	0.09 ± 0.00^a^	–	–	–
8	Cyclopentanol, 1-methyl-	1462-03-9	2465	1.25	0.30 ± 0.06^c^	0.61 ± 0.04^ab^	0.88 ± 0.05^a^	0.17 ± 0.02^e^	0.32 ± 0.02^c^	0.25 ± 0.07^cd^
9	Ethanol	64–17-5	1983	1.81	0.98 ± 0.07^d^	1.67 ± 0.05^a^	1.25 ± 0.13^bc^	1.10 ± 0.02^bc^	1.51 ± 0.05^ab^	1.75 ± 0.13^a^
10	Ethylene glycol	107–21-1	2243	1.03	2.78 ± 0.05^a^	2.53 ± 0.23^b^	2.55 ± 0.09^b^	2.43 ± 0.11^b^	2.19 ± 0.23^bc^	1.91 ± 0.14^d^
11	Cis-4-Cyclopentene-1,3-diol	29,783–26-4	1644	1.38	–	–	–	0.10 ± 0.01^b^	0.23 ± 0.05^a^	–
12	Pentanol	71–41-0	1674	1.17	–	0.87 ± 0.10^b^	1.59 ± 0.02^a^	–	–	–
13	2,7-Dimethyl-1-octanol	15,250–22-3	1678	1.73	0.05 ± 0.01^a^	–	–	–	–	0.42 ± 0.02^a^
14	1-Hexanol	111–27-3	1824	1.19	1.09 ± 0.03^a^	0.95 ± 0.03^ab^	–	0.46 ± 0.06^cd^	0.69 ± 0.05^c^	0.65 ± 0.06^c^
15	3-Ethyl-4-methylpentan-1-ol	38,514–13-5	2023	1.24	–	–	0.48 ± 0.06^a^	–	0.12 ± 0.01^ab^	0.09 ± 0.01^b^
16	Hexahydrofarnesol	6750-34-1	2332	1.24	0.23 ± 0.03^a^	–	–	0.05 ± 0.00^b^	–	–
17	4-Ethyl-1-octyn-3-ol	5877-42-9	2267	1.34	0.08 ± 0.02^a^	–	–	–	–	–
18	2-Hexyl-1-decanol	2425-77-6	1441	1.59	–	0.22 ± 0.07^ab^	0.27 ± 0.05^a^	–	–	–
19	1-Octanol,2-butyl-	3913–02-8	1475	1.13	–	0.08 ± 0.00^c^	0.26 ± 0.01^ab^	0.24 ± 0.04^ab^	0.29 ± 0.06^ab^	0.41 ± 0.01^a^
20	1-Hexadecanol,2-methyl-	2490-48-4	1480	1.53	0.17 ± 0.02^ab^	0.18 ± 0.01^ab^	–	0.21 ± 0.03^a^	–	–
21	9E-Hexadecen-1-ol	64,437–47-4	1456	1.22	0.15 ± 0.02	–	0.05 ± 0.00b	0.05 ± 0.01b	0.10 ± 0.00a	0.19 ± 0.03^a^
22	2-ethyldodecan-1-ol	19,780–33-7	1514	1.47	0.71 ± 0.01^a^	0.49 ± 0.09^b^	0.23 ± 0.04^d^	0.36 ± 0.01^bc^	0.73 ± 0.05^a^	0.35 ± 0.06^bc^
23	1-Nonadecanol	1454-84-8	1522	1.34	0.28 ± 0.08^a^	–	–	–	–	–
24	3-octen-2-ol	76,649–14-4	1522	1.61	0.06 ± 0.01^d^	0.20 ± 0.02^bc^	1.00 ± 0.09^a^	0.26 ± 0.07^bc^	0.26 ± 0.03^bc^	0.42 ± 0.04^b^
25	1-Nonen-4-ol	35,192–73-5	1548	1.43	0.10 ± 0.02^bc^	0.11 ± 0.01^bc^	0.13 ± 0.02^b^	0.13 ± 0.02^b^	0.23 ± 0.04^a^	0.14 ± 0.03^b^
26	1-Octanol	111–87-5	1612	1.46	0.08 ± 0.01^ab^	–	0.06 ± 0.01^b^	0.12 ± 0.02^a^	–	–
**Ketone (17)**										
1	Cyclobutyl phenyl ketone	5407-98-7	1559	1.79	0.48 ± 0.06^bc^	0.18 ± 0.03^c^	0.50 ± 0.09^b^	0.97 ± 0.05^a^	0.43 ± 0.20^bc^	0.69 ± 0.10^b^
2	Ethanedione, (4-methylphenyl)phenyl-	2431-00-7	1594	1.10	0.13 ± 0.01^bc^	0.25 ± 0.04^ab^	0.12 ± 0.03^bc^	0.16 ± 0.02^b^	0.31 ± 0.04^a^	0.20 ± 0.01^ab^
3	2(3H)-Benzofuranone, 3a,4,5,6-tetrahydro-3a,6,6-trimethyl-	16,778–26-0	2117	2.03	0.10 ± 0.01^ab^	–	–	–	0.12 ± 0.05^a^	–
4	4-Hydroxy-2-methylacetophenone	875–59-2	2225	1.02	0.56 ± 0.04^b^	0.54 ± 0.08^b^	1.00 ± 0.04^a^	0.50 ± 0.06^b^	0.08 ± 0.03^d^	0.21 ± 0.07^c^
5	2,2-Dianilinoacetophenone	66,749–88-0	2357	1.75	0.15 ± 0.02^c^	0.50 ± 0.05^b^	1.00 ± 0.02^a^	0.10 ± 0.01^cd^	0.10 ± 0.02^cd^	0.20 ± 0.03^bc^
6	Ethanone, 1,1′-(1,3-phenylene)bis-	6781-42-6	2372	1.25	0.10 ± 0.01^de^	0.54 ± 0.08^c^	2.76 ± 0.12^a^	0.22 ± 0.01^d^	0.10 ± 0.02^de^	1.05 ± 0.13^b^
7	Phenyl 2-(2′-furanyl)cyclopropyl ketone	N/A	2406	1.72	0.17 ± 0.04^b^	–	–	0.23 ± 0.05^a^	–	–
8	(E)-Oct-3-en-2-one	18,402–82-9	2037	2.20	0.19 ± 0.01^a^	0.08 ± 0.02^c^	0.04 ± 0.01^cd^	–	0.06 ± 0.01^c^	0.10 ± 0.00^ab^
9	2-Nonanone	821–55-6	2151	1.11	0.29 ± 0.02^a^	0.21 ± 0.18^a^	0.11 ± 0.03^ab^	0.07 ± 0.02^c^	0.16 ± 0.02^ab^	0.15 ± 0.01^ab^
10	3-Nonen-2-one	14,309–57-0	2192	1.72	0.10 ± 0.02^a^	0.10 ± 0.01^d^	–	0.09 ± 0.03^b^	0.05 ± 0.01^bc^	0.11 ± 0.02^a^
11	5-Ethyl-6-methyl-3-hepten-2-one	57,283–79-1	2200	1.65	0.08 ± 0.04^ab^	0.10 ± 0.04^a^	0.10 ± 0.05^a^	0.10 ± 0.05^a^	0.10 ± 0.03^a^	0.10 ± 0.04^a^
12	2-Decanone	693–54-9	2331	1.82	0.20 ± 0.04^c^	–	0.05 ± 0.02^d^	0.50 ± 0.04^a^	0.05 ± 0.04^d^	0.40 ± 0.02^ab^
13	neryl acetone	3879-26-3	2531	1.30	0.79 ± 0.04^a^	0.40 ± 0.04^ab^	0.39 ± 0.05b^c^	0.59 ± 0.06^ab^	0.31 ± 0.05^bc^	0.41 ± 0.06^ab^
14	2-Hydroxychalcone	644–78-0	2590	1.85	0.06 ± 0.01^b^	–	–	–	0.19 ± 0.05^a^	–
15	2-Heptanone	110–43-0	1293	1.17	0.66 ± 0.04^bc^	0.74 ± 0.08^b^	1.98 ± 0.04^a^	0.54 ± 0.06^cd^	0.50 ± 0.03^cd^	0.62 ± 0.07^bc^
16	3-Octanone, 2-methyl-	923–28-4	1320	1.60	0.15 ± 0.02^d^	0.40 ± 0.05^b^	1.65 ± 0.04^a^	0.24 ± 0.01^bc^	0.20 ± 0.02^bc^	0.33 ± 0.03^b^
17	Phytone	502–69-2	1325	1.57	0.26 ± 0.04^a^	–	0.10 ± 0.02^bc^	0.13 ± 0.04^b^	0.10 ± 0.04^bc^	0.12 ± 0.06^b^
**Olefins (8)**										
1	(Halogen)-Limonene	138–86-3	1218	1.02	0.20 ± 0.03^a^	0.17 ± 0.02^ab^	0.15 ± 0.03^ab^	0.05 ± 0.00^c^	0.22 ± 0.01^a^	0.05 ± 0.00^c^
2	1,3,5-Cycloheptatriene, 7,7-dimethyl-	2,066,483	1251	2.01	0.24 ± 0.09^a^	0.22 ± 0.01^ab^	0.20 ± 0.02^ab^	0.16 ± 0.04^bc^	0.12 ± 0.01^cd^	0.18 ± 0.01^bc^
3	Bicyclo[4.1.0]hept-2-ene, 3,7,7-trimethyl-, (1S-cis)-	4497-92-1	1305	1.67	0.10 ± 0.01^a^	–	–	–	–	–
4	1-Nonene, 4,6,8-trimethyl-	54,410–98-9	1679	1.28	0.17 ± 0.02^b^	–	0.61 ± 0.09^a^	–	–	–
5	Dispiro[2.2.2.2]deca-4,9-diene	36,262–33-6	1802	1.60	0.49 ± 0.03^a^	–	0.09 ± 0.03^b^	–	–	–
6	(E)-1-(2,3,6-trimethylphenyl)buta-1,3-diene (TPB, 1)	N/A	1950	1.35	0.30 ± 0.04^ab^	0.17 ± 0.03^c^	0.10 ± 0.03^cd^	0.22 ± 0.03^bc^	0.11 ± 0.04^cd^	0.41 ± 0.07^a^
7	2-Octene	111–67-1	1410	1.48	–	0.12 ± 0.04^b^	1.35 ± 0.01^a^	0.08 ± 0.01^bc^	0.11 ± 0.01^b^	0.08 ± 0.02^bc^
8	4,5-Nonadiene	821–74-9	2709	1.11	–	–	0.11 ± 0.01^b^	0.45 ± 0.04^a^	–	–
**Esters (13)**										
1	Thioglycolic acid, *tert*-butyldimethylsilyl ester	N/A	1485	1.41	–	–	–	0.16 ± 0.01^ab^	0.21 ± 0.02^a^	–
2	Oxalic acid, isobutyl pentyl ester	N/A	1539	1.82	0.05 ± 0.02^a^	–	–	–	–	0.05 ± 0.01^a^
3	3-Phenylpropionic acid, 4-cyanophenyl ester	N/A	1609	1.75	0.12 ± 0.02^a^	–	–	–	–	–
4	Butyric acid, 2-phenyl-, dec-2-yl ester	N/A	1611	1.59	0.04 ± 0.02^c^	0.03 ± 0.01^cd^	0.09 ± 0.02^ab^	0.13 ± 0.08^a^	0.04 ± 0.01^c^	–
5	Benzeneacetic acid, .alpha.-oxo-, methyl ester	15,206–55-0	1688	1.34	0.24 ± 0.04^a^	–	–	0.07 ± 0.03^b^	–	–
6	(1*R*,3*R*,4*S*,5*S*)-1-Isopropyl-4-methylbicyclo[3.1.0]hexan-3-yl acetate-rel	72,747–24-1	1719	1.68	0.32 ± 0.04^a^	–	–	0.07 ± 0.00^b^	–	0.03 ± 0.01^bc^
7	Oxalic acid, allyl pentadecyl ester	N/A	1723	1.59	0.20 ± 0.02^a^	–	–	0.06 ± 0.00^c^		0.18 ± 0.03^ab^
8	4-Methylbenzyl salicylate	N/A	1754	1.25	0.03 ± 0.02^cd^	0.08 ± 0.01^ab^	0.09 ± 0.02^ab^	0.13 ± 0.04^a^	0.04 ± 0.02^c^	–
9	Sulfurous acid, butyl nonyl ester	N/A	1778	1.24	0.08 ± 0.04^cd^	0.08 ± 0.03^cd^	0.13 ± 0.03^b^	0.65 ± 0.03^a^	0.12 ± 0.04^b^	0.10 ± 0.04^bc^
10	3-Phenylpropyl benzoate	60,045–26-3	2376	1.31	–	0.04 ± 0.02^a^	0.03 ± 0.00^ab^	–	–	–
11	1-Naphthalenemethyl naphthalene-2-carboxylate	86,328–63-4	2404	1.15	0.07 ± 0.03^ab^	0.07 ± 0.02^ab^	0.04 ± 0.01^bc^	0.05 ± 0.03^bc^	0.06 ± 0.01^bc^	0.08 ± 0.01^a^
12	Methyl trans-2-triethylsilyl-cyclopropane-1-carboxylate	N/A	2454	1.04	0.07 ± 0.01^b^	–	–	0.56 ± 0.03^a^	–	0.04 ± 0.02^bc^
13	Cyclohexyl salicylate	N/A	2461	1.78	0.05 ± 0.02^a^	–	–	–	–	0.04 ± 0.02^ab^
**Other (14)**										
1	Benzene, 1,1′-(1,5-hexadiene-1,6-diyl)bis-	4439–45-6	1465	1.47	0.22 ± 0.03^bc^	0.21 ± 0.02^bc^	0.26 ± 0.02^bc^	0.41 ± 0.03^ab^	0.06 ± 0.02^d^	0.43 ± 0.05^a^
2	1-Chloro-2-trihexylsilyloxybenzene	N/A	1555	1.68	–	0.02 ± 0.01^a^	0.02 ± 0.00^a^	–	–	–
3	1,3-Dimethyl-pyridinium chloride	N/A	1612	1.30	–	–	–	0.24 ± 0.02^bc^	0.30 ± 0.04^a^	0.29 ± 0.05^ab^
4	Benzene, (1-methyl-1-propylpentyl)-	54,932–91-1	1673	1.67	0.42 ± 0.01^ab^	–	–	–	–	0.59 ± 0.06^a^
5	Cyclobutylcarboxamide, *N*-methyl-*N*-phenyl-	N/A	1684	1.46	0.23 ± 0.03^cd^	1.15 ± 0.13^ab^	2.98 ± 0.21^a^	1.13 ± 0.12^ab^	0.02 ± 0.01^e^	0.49 ± 0.14^c^
6	2,6-Dimethyl-1,3,6-heptatriene	928–67-6	1751	1.15	0.13 ± 0.03^ab^	0.06 ± 0.02^c^	1.54 ± 0.01^a^	0.05 ± 0.03^cd^	0.05 ± 0.01^cd^	0.09 ± 0.01^bc^
7	Benzene, 1-(2-bromoethyl)-4-methyl-	6529-51-7	1817	1.53	0.23 ± 0.01^e^	2.04 ± 0.05^a^	1.47 ± 0.04^b^	0.31 ± 0.02^c^	0.26 ± 0.01^cd^	0.28 ± 0.02^cd^
8	Benzene, 1-(1-formylethyl)-4-(1-buten-3-yl)-	N/A	1865	2.18	0.34 ± 0.03^bc^	0.22 ± 0.02^de^	0.47 ± 0.03^ab^	0.27 ± 0.04^cd^	0.66 ± 0.03^a^	0.65 ± 0.06^a^
9	Acetophenone, 4-nitro-, 6-pyrazolylcarbonylhydrazone	N/A	2102	1.83	–	0.06 ± 0.01^ab^	–	–	0.08 ± 0.01^a^	–
10	3-Methyl-2-(3,7,11-trimethyldodecyl) furan	166,773–55-3	2127	1.31	0.33 ± 0.05^ab^	–	–	0.08 ± 0.01^cd^	0.11 ± 0.01^bc^	0.52 ± 0.08^a^
11	Phenol, 2-methoxy-6-(2-propenyl)-	579–60-2	2194	1.20	0.06 ± 0.01^c^	0.12 ± 0.02^ab^	0.18 ± 0.02^ab^	–	0.05 ± 0.03^cd^	0.23 ± 0.03^a^
12	6-Phenyl-5,6-dihydro-5,6-azaboruracil	78,594–53-3	2194	1.69	0.15 ± 0.04^d^	0.50 ± 0.04^a^	0.33 ± 0.06^ab^	0.48 ± 0.04^a^	0.20 ± 0.05^bc^	0.28 ± 0.04^bc^
13	3-Butenyl adipate	N/A	2280	1.00	0.16 ± 0.02^d^	0.25 ± 0.02^bc^	0.40 ± 0.04^b^	0.56 ± 0.02^b^	1.76 ± 0.07a	0.24 ± 0.04^bc^
14	2-acetyl-1-pyrroline	85,213–22-5	1751	1.74	1.16 ± 0.03^a^	–	0.06 ± 0.01^c^	1.12 ± 0.03^ab^	1.10 ± 0.06^ab^	1.14 ± 0.10^ab^

Note: “-” indicates that the compound was not detected; different letters indicate that the compound was significantly different among different rice varieties (*P*﹤0.05).

The ratio of the peak area of each compound to the total peak area of all compounds was taken as the relative content of the compound, and the relative contents of volatile compounds in different varieties of rice were further analyzed (Table 2). The relative contents of various volatile substances in different varieties are, from high to low, alkanes (10.01 % ∼ 25.90 %), aldehydes (16.00 % ∼ 70.45 %), alcohols (7.56 % ∼ 15.97 %), ketones (2.86 % ∼ 9.80 %), olefins (0.56 % ∼ 2.61 %), acid esters (0.30 % ∼ 1.88 %), and others (3.43 % ∼ 5.71 %). In addition, there were significant differences in the content of volatile substances among different rice varieties. The proportion of alkanes in the WC54, ZKF5 and SJ18 rice varieties was relatively large, at 25.90 %, 17.49 % and 16.31 % respectively; while the proportion of alkanes in the DN425, LY11 and LY16 rice varieties was relatively small, at 12.34 %, 11.78 % and 10.01 % respectively. The proportion of aldehyde substances in rice varieties DN425, LY11, LY16 and WC54 was relatively large, at 70.45 %, 62.89 %, 54.48 % and 54.35 % respectively; while the proportion of aldehyde substances in rice varieties ZKF5 and SJ18 was relatively small, at 17.49 % and 16.00 % respectively. It can be seen that alkanes and aldehydes are the main different volatile compounds in different rice varieties, which may be affected by environmental factors such as light, temperature and humidity, resulting in differences in the types of alkane and aldehyde compounds in different rice varieties (Lim et al., 2018b; Wang et al., 2025). For example, Ch et al. (2021) found that the content of five hydrocarbon compounds in Chinese rice was higher, and used them as key volatile compounds to distinguish Chinese rice from Vietnamese and Indian rice. Therefore, by analyzing the differences in volatile compounds in rice from different origins, it can be used to distinguish different geographical indication rice.

Most of the different volatile compounds among different varieties of rice after screening were aldehydes and alkanes, which is consistent with the results of Asimi et al. (2022), who also confirmed that aldehyde compounds were the key aroma markers for distinguishing rice varieties. In addition, the differences in the types of volatile compounds detected by HS-SPM*E*-GC–MS are consistent with previous studies by E-nose, indicating that these volatile compounds had an important influence on the flavor characteristics of rice. For example, hexanal and nonaldehyde substances, such as fruit and floral aroma, are the main contributors to rice aroma (Zeng et al., 2007; Bryant & Mcclung, 2011a). This differential analysis is of great significance for the flavor characteristics of different varieties of rice, the selection and breeding of high-quality rice varieties, and meeting consumers' diverse needs for rice flavor.

#### 3.3.2 Principal component analysis of volatile components in different varieties of rice

Principal component analysis (PCA) was used to extract characteristic values from the fingerprint information of 148 common volatile components in different varieties of rice. The key characteristic volatile components were screened according to the maximum contribution degree of each principal component factor (positive contribution value and negative contribution value). These key characteristics of volatile components are of great significance for distinguishing different varieties of rice. For example, Chen et al., 2023 screened out characteristic volatile components such as ethanol, 1-hexanol, hexanal, heptaldehyde, and nonanal by PCA. The content differences of these components in different varieties of rice may become a key factor in distinguishing varieties. The amount of data analyzed was reduced to facilitate the classification and discrimination of different rice varieties. The contribution rate of principal component 1 was 31.5 %, the contribution rate of principal component 2 was 25.6 %, and the total cumulative contribution rate was 57.1 %. This result shows that the two principal components extracted can effectively retain the original characteristic data and meet the reliability requirements of data analysis. Based on this, this study used the first two principal components to construct discriminant model for systematically comparing the overall volatile compound characteristics of rice samples of different varieties.

**Fig. 3:**
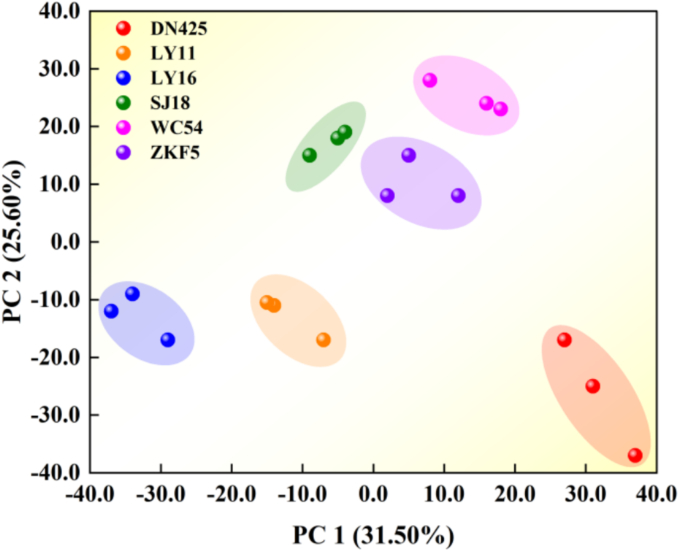
PCA plots of volatile components of different varieties of rice in Wuchang.

As shown in Fig. 3, the spatial distribution of different rice varieties can be intuitively displayed, and rice samples of different varieties can be effectively distinguished. Among them, the first principal component mainly reflects the relevant information of substances such as alkanes and aldehydes, which occupy an important position in the volatile flavor components, indicating that they have a significant impact on the overall flavor characteristics of rice. The second principal component mainly reflects the relevant information of alcohols, ketones, olefins, acid esters and other substances. Although these substances account for a relatively low proportion in the total volatile components, they also play an important role in the flavor characteristics of rice. For example, in rice varieties WC54, SJ18, ZKF5, LY16, LY11, and DN425, alkanes and aldehydes are the main contributors to aroma compounds. WC54 (35) and ZKF5 (33) rice varieties had a larger number of alkane types, while DN425 (24) rice variety had the fewest. The number of aldehyde types in LY16 (21) and LY11 (22) rice varieties was relatively large, DN425 (16) rice variety had the fewest. These differences might have been due to factors such as soil and climatic conditions, which affected the synthesis and accumulation of volatile components, thus forming their unique aroma characteristics (Hu et al., 2020; Hui et al., 2022). In conclusion, the principal component analysis method can not only effectively identify rice varieties and evaluate their quality, but also accurately distinguish the differences in volatile component composition of different rice varieties by screening key characteristic volatile components, providing a reliable basis for the identification and quality analysis of rice varieties.

### 3.4 Classification and discrimination model for different varieties of rice based on volatile compounds

#### 3.4.1 Establishment of discriminant models

**Fig. 4:**
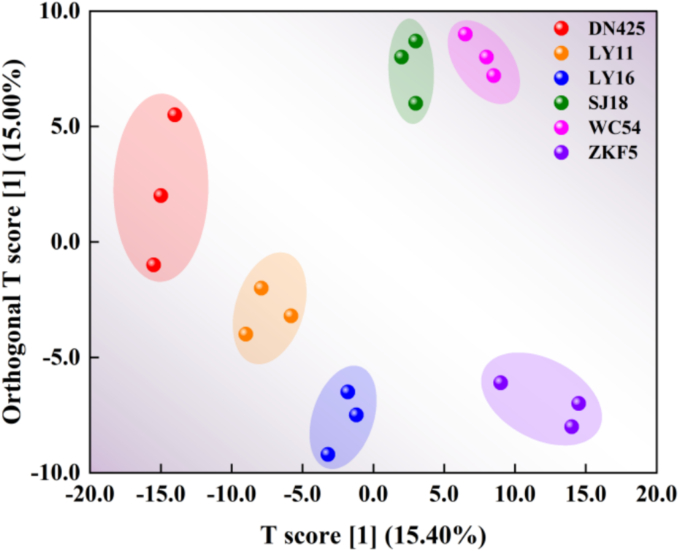
OPLS-DA plots for volatile compounds in different varieties of rice in Wuchang.

OPLS-DA was used to further analyze the compounds with significant differences among different rice samples. As shown in Fig. 4, different rice samples could be well distinguished according to volatile compounds, and the correct identification rate is high.

#### 3.4.2 Verification of discriminant models

**Fig. 5:**
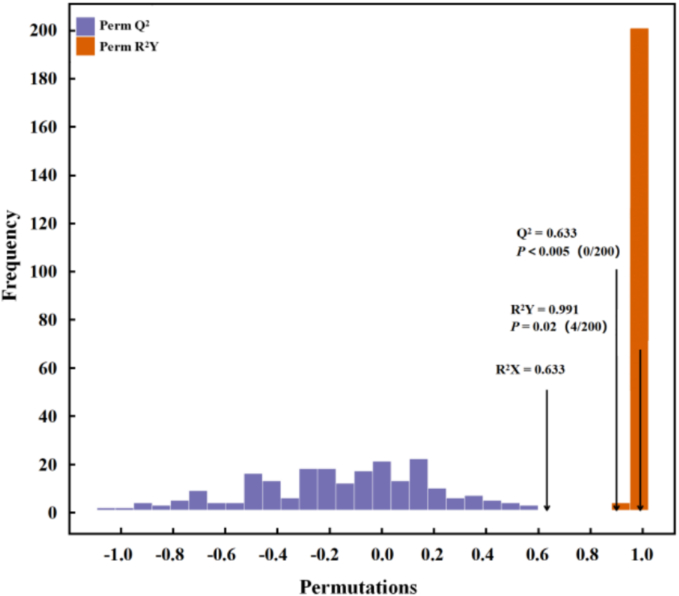
OPLS-DA verification diagram for volatile compounds in different varieties of rice in Wuchang city.

The prediction parameters of the OPLS-DA evaluation model include R^2^X, R^2^Y and Q^2^, where R^2^X and R^2^Y represent the interpretation rate of the built model to the X matrix and Y matrix respectively, and Q^2^ represents the prediction ability of the model. The closer the three indexes are to 1, the more stable and reliable the model is. Q^2^ > 0.5 can be regarded as an effective model, Q^2^ > 0.9 as an excellent model (Wiklund et al., 2008). Fig. 5 showed the OPLS-DA verification diagram. The well-explained variance (R^2^Y = 0.991) and the cross-validated predictive power (Q^2^ = 0.900) in the figure demonstrated the stability and reliability of the model, making it suitable for further analysis and prediction.

### 3.5 Analysis and identification of aroma components of different rice varieties based on GC-O

The aroma components of six rice varieties were systematically analyzed and identified. As shown in Table 3, a total of 36 volatile aroma components were detected, among which aldehyde compounds showed significant characteristics in all six varieties. Comparative analysis of aroma intensity revealed that the overall aroma intensity of WC54 variety was significantly higher than that of other rice varieties. WC54 had large amount of aroma active substances, including esters, aldehydes and ketones. This was consistent with previous research results (Kazunao et al., 2019; Xua et al., 2020). Based on the volatile compounds and sensory characteristics of different aromatic rice varieties, it was found that rice had a high content of esters, aldehydes and ketones, which were the main sources of its fruity and creamy flavors.

**Table 3:** Identification of rice aroma components.

NO.	Aroma characteristic	Aroma intensity					
		DN425	LY11	LY16	SJ18	WC54	ZKF5
1	Fruity	1	0	0	0	0	0
2	Faint scent	0	0	0	1	1	0
3	Taste of alcohol	0	1	2	1	1	1
4	Wood and fruit aromas	0	0	0	0	1	1
5	Faint scent	2	2	2	2	2	2
6	Aroma of grass	3	3	1	2	3	1
7	Fruity	2	0	2	0	2	1
8	Fruity	1	1	1	1	2	2
9	Smoky taste	1	1	1	1	1	1
10	Sweet and fragrant	0	0	1	1	1	1
11	Rosette	1	0	0	0	1	1
12	Breadiness	2	2	2	1	3	2
13	Grass, citrus fragrance	1	1	1	1	1	1
14	Fruity	1	1	1	1	2	1
15	Fruity	2	2	1	1	1	2
16	Popcorn taste	3	3	2	2	4	2
17	Herb fragrance	2	1	1	1	2	1
18	Sweet and nutty	2	2	2	2	2	2
19	Bitter almond	2	2	2	2	2	2
20	Bitter taste	1	1	1	1	1	1
21	Citrus, soapy	3	1	2	2	3	2
22	Grassy, fatty taste	0	0	0	0	0	1
23	Mushroom, earthiness	2	2	2	1	2	1
24	Acidity	1	1	0	0	0	0
25	Aroma of cream	1	2	1	2	2	1
26	Nutty, bitter taste	1	2	1	1	1	1
27	Malt flavor	1	1	2	1	1	2
28	Fatty taste	3	2	2	3	3	2
29	Pungent smell	2	2	1	1	1	1
30	Floral scent	1	0	0	0	1	0
31	Rose, citrus	2	1	2	2	2	1
32	Nutty	1	1	1	1	1	1
33	Medicated incense	1	0	0	0	0	0
34	Stink	0	1	0	0	0	0
35	Fruity	2	3	2	1	3	2
36	Vanilla flavor	1	2	2	1	2	1

### 3.6 Differential analysis of volatile compounds between WC54 and other rice varieties

**Fig. 6:**
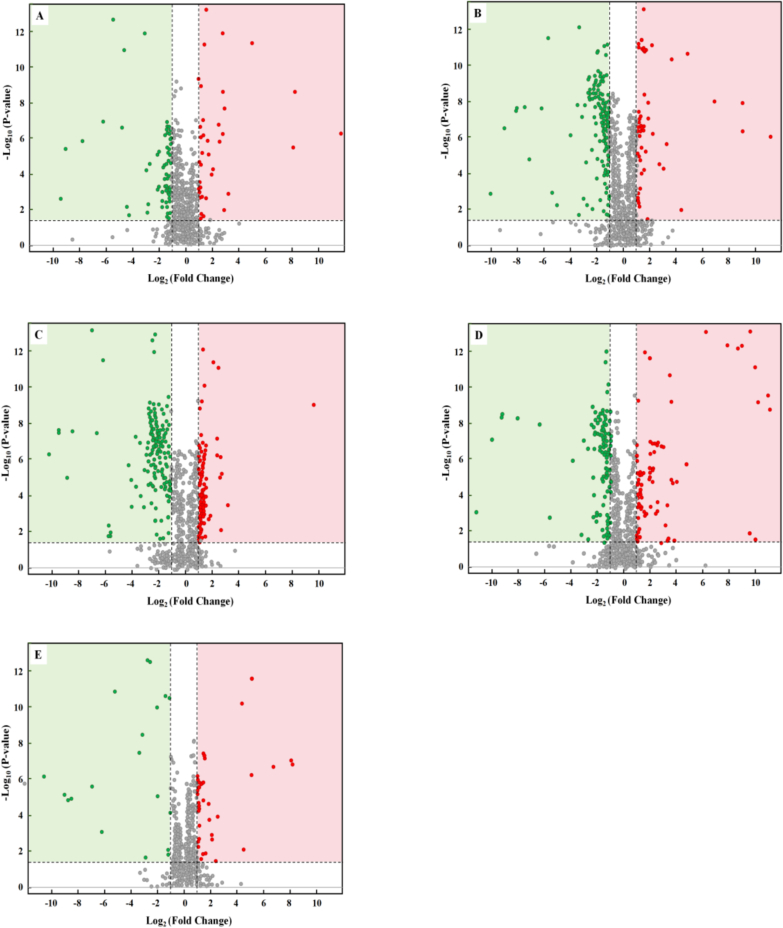
Volcanic of WC54 compared with other 5 varieties of rice. (A: WC54 vs. ZKF5; B: WC54 vs. SJ18; C: WC54 vs. LY16; D: WC54 vs. LY11; E: WC54 vs. DN425).

Volcano Plot is mainly used to show the relative content difference of metabolites in two groups of samples and the statistical significance of the difference (Metayer et al., 2002). In order to illustrate the differences in volatile compounds between WC54 and the other five rice varieties, the differential volatile compounds detected by HS-SPME-GC–MS were compared, and a volcano plot of WC54 and the other five rice varieties was drawn, as shown in Fig. 6.

Each point in the volcano plot represents a metabolite, where green points represent down-regulated differential compounds, red points represent up-regulated differential compounds, and gray points represent compounds that were detected but not significantly different. As shown in Fig. 6, the most different up-regulated compound among WC54, DN425, and SJ18 rice was tridecane, while the most different volatile compound between WC54 and ZKF5 rice was pentadecane. Both tridecane and pentadecane belong to the class of long-chain alkanes, and their synthesis is usually closely related to the fatty acid metabolism pathway of plants. The synthesis of long-chain alkanes (such as tridecane and pentadecane) can be achieved through biosynthetic pathways in plants, which mainly involve fatty acid metabolism and the catalysis of related enzyme systems. Specifically, fatty acids in plant cells can be extended and modified through a series of enzymatic reactions to eventually form long-chain alkanes (Huang et al., 2020; Wen et al., 2025); The volatile compound with the greatest difference between WC54 and LY11 rice was 2-acetyl-1-pyrroline. It is the key component of the unique flavor of fragrant rice, and its content directly determines whether the rice has the typical popcorn or nutty aroma of fragrant rice (Buttery et al., 1983; Wei et al., 2017). LY11 rice is a non-aromatic rice, which is in sharp contrast to WC54 rice. This difference may be related to the expression or functional status of the *BADH*_*2*_ gene. Mutation or deletion of the *BADH*_*2*_ gene leads to the accumulation of 2-AP, thus forming the flavor of aromatic rice (Sansenya et al., 2018); The volatile compound with the greatest difference between WC54 and LY16 rice was decanal, which may be related to factors such as light conditions and soil fertilizer during growth. For example, changes in light conditions can regulate plant photosynthesis and secondary metabolism, thereby affecting the oxidative decomposition of fatty acids and producing more aldehyde volatile compounds (Bryant and Mcclung, 2011b; Gao et al., 2021). In addition, the nutrients in fertilizers (such as nitrogen, phosphorus, potassium, etc.) can regulate the growth and metabolic processes of plants and increase the synthesis of fatty acids, thereby indirectly affecting the content of aldehyde compounds (Yaakob et al., 2021).

In addition, based on the up-regulated and down-regulated differential compounds and the VIP values between WC54 and five different rice varieties, the top 10 volatile compounds with the greatest differences were screened out, namely tridecane, decanal, hexanal, nonanal, tetradecane, 2-acetyl-1-pyrroline, heptaldehyde, pristane, pentadecane, and 3-octen-2-one. However, although compounds such as tridecane and tetradecane are not the main aroma components, their presence may affect the overall flavor and taste of rice. Therefore, the differences in these volatile compounds can also be used as important indicators for quality evaluation (Wang et al., 2025).

## 4 Conclusion

The volatile odor characteristics of six rice varieties grown in five regions were systematically analyzed using electronic nose, olfactometer and HS-SPME-GC–MS techniques. The results showed that although the electronic nose technology could achieve preliminary differentiation, the HS-SPME-GC–MS technology provided more accurate and comprehensive analysis results, and a total of 148 volatile compounds with significant odor characteristics were identified. Orthogonal partial least squares discriminant analysis (OPLS-DA) based on GC–MS data effectively revealed the differential characteristics among different varieties, especially clarifying the key volatile markers that distinguished the WC54 variety from the other five varieties. Therefore, this study provides a scientific basis for the protection and identification of geographical indication rice varieties by accurately analyzing the volatile components of rice varieties that are similar in appearance but different in variety.

## CRediT authorship contribution statement

**Lili Qian:** Writing – review & editing, Methodology, Conceptualization. **Mingming Chen:** Writing – original draft, Validation, Software, Formal analysis. **Yan Song:** Software, Methodology. **Tao Zhang:** Methodology, Investigation, Conceptualization. **Xingquan Liu:** Methodology, Investigation, Conceptualization. **Guoxin Zhou:** Methodology, Investigation, Conceptualization. **Hongyan Liu:** Writing – review & editing, Funding acquisition. **Feng Zuo:** Writing – review & editing, Supervision.

## Funding

This work was supported by the Research and Demonstration of Northeast Japonica Rice Harvesting and Post-harvest Storage Loss Reduction Technology (2023YFD2301604–4), the Sichuan Science and Technology Program (2023ZYD0272), the Local Financial Funds of National Agricultural Science and Technology Center (NASC2023ST04 and NASC2024KY22), and the 10.13039/501100012421Agricultural Science and Technology Innovation Program (ASTIP2025-34-IUA-09).

## Declaration of competing interest

The authors declare that they have no known competing financial interests or personal relationships that could have appeared to influence the work reported in this paper.

## Data Availability

The data that has been used is confidential.
